# Prevalence and Therapeutic Challenges of Fungal Drug Resistance: Role for Plants in Drug Discovery

**DOI:** 10.3390/antibiotics9040150

**Published:** 2020-03-31

**Authors:** Lewis Marquez, Cassandra L. Quave

**Affiliations:** 1 Molecular and Systems Pharmacology, Emory University, Atlanta, GA 30322, USA; lewis.marquez@emory.edu; 2 Department of Dermatology and Center for the Study of Human Health, Emory University, Atlanta, GA 30322, USA

**Keywords:** antifungal resistance, *Candida auris*, multidrug resistant, ethnobotany, traditional medicine, drug discovery

## Abstract

Antimicrobial resistance is a global issue that threatens the effective practice of modern medicine and global health. The emergence of multidrug-resistant (MDR) fungal strains of *Candida auris* and azole-resistant *Aspergillus fumigatus* were highlighted in the Centers for Disease Control and Prevention’s (CDC) 2019 report, *Antibiotic Resistance Threats in the United States*. Conventional antifungals used to treat fungal infections are no longer as effective, leading to increased mortality. Compounding this issue, there are very few new antifungals currently in development. Plants from traditional medicine represent one possible research path to addressing the issue of MDR fungal pathogens. In this commentary piece, we discuss how medical ethnobotany—the study of how people use plants in medicine—can be used as a guide to identify plant species for the discovery and development of novel antifungal therapies.

## 1. Introduction

Drug resistance among fungal pathogens is continuing to develop into an increasingly serious threat to public health and health-care systems worldwide. No longer susceptible to conventional antifungal drugs, these multidrug-resistant fungal pathogens make the development of novel antifungals a public health imperative. Using the traditional knowledge of plants as medicines, or ethnobotanical knowledge, we can incorporate medically relevant plant species into the drug discovery process. This is because as far back as historical and archaeological records show, humans have used plants in the treatment of myriad diseases and ailments. The ethnobotanical knowledge found within each country and culture is a source of medical insight that has been largely overlooked by the conventional pharmaceutical industry; this knowledge can be used to address the issue of drug-resistant microbes.

## 2. New Emerging Fungal Threats

The especially hardy and persistent fungal pathogen *Candida auris* was one of the newly emerging urgent threats highlighted by the CDC’s 2019 report *Antibiotic Resistance Threats in the United States* [1]. Discovered in 2009, *C. auris* has been responsible for outbreaks in health-care facilities worldwide [2,3,4,5,6,7]. There was a 318% increase in the number of reported *C. auris* infections in 2018 compared to rates from 2015–2017 [1]. Multiple studies examining *C. auris* infections from hospitals in India, Colombia, and New York found that 58–76% of these lead to invasive blood stream infections [2,8,9]. 

With more than 34,000 cases and 1700 deaths annually, drug-resistant *Candida* species (such as *C. albicans*, *C. glabrata*, *C. parapsilosis*, *C. tropicalis*, *C. krusei*) were highlighted by the CDC’s 2019 report as a serious threat [1]. Their designation as a serious threat is in part due to the high likelihood of inherent and acquired resistance among the related *Candida* spp. For example, *C. krusei* was found to have intrinsic resistance to fluconazole as far back as 1989 [10]. Acquired resistance can also develop after antifungal exposure, and troubling cases of cross-resistance to the azoles and echinocandins have been observed among *Candida* spp., highlighting the real threat posed by drug resistance.

Another emerging fungal threat that was placed on the CDC’s watch list is *Aspergillus fumigatus*. Development of drug resistance in this species has been linked to environmental fungicide exposure and this pathogen is now resistant to fluconazole [11,12]. The development of multidrug resistance in this species raises cause for future concerns.

*Cryptococcus neoformans* is another deadly fungal pathogen and, while infection is rare in healthy individuals, there are roughly 220,000 cases of cryptococcal meningitis in immunocompromised individuals worldwide and 181,000 deaths a year [13]. There has been an uptick in incidences of increased drug resistance seen in clinical strains of *C. neoformans,* which may exacerbate the already high mortality rate [14].

Absent from the CDC’s 2019 report is any mention of dermatophytes. These are fungal pathogens that cause superficial infections of the skin, hair, and nails. While dermatophytes do not often cause life-threatening infections, their prevalence among the population has been estimated at nearly one billion cases globally [15]. A study examining trends in dermatophyte infections in Poland from 2011 to 2016 found that 1600 patients, nearly 15% of all patients examined, presented with a form of a superficial fungal infection [16]. Cultures from these patients identified nearly 1800 different strains, of which more than 900 were dermatophytes. A separate study examining antifungal consumption rates in Belgium found that superficial fungal infections caused by dermatophytes accounted for nearly 25% of the disease burden as measured by disability-adjusted life years, or years of life lost to illness [17].

The prevalence of drug resistance among fungal pathogens is becoming increasingly common and is poised to emerge as a public health crisis in the near future. Tackling the issue of antifungal resistance will be difficult using the current set of chemical tools. There is an immediate need for the discovery and development of new drugs that display broad-spectrum activity and can effectively target drug-resistant fungal strains.

## 3. Antifungal Drugs

### 3.1. Challenges Facing Current Antifungals 

Antifungal drugs produce their effect by exploiting differences between human and fungal cells at two major targets, the cell membrane and the fungal cell wall. Three major classes of antifungals are typically prescribed: polyenes, azoles, and echinocandins. Amphotericin B and other polyenes produce pores in the fungal cell membrane leading to cell death [18]. Fluconazole and other azoles inhibit C14-α sterol demethylase and destabilize the structural integrity of the fungal cell membrane [18]. Caspofungin and other echinocandins disrupt fungal cell wall synthesis by inhibiting the synthesis of β-1,3-D-glucan [18]. 

The major challenge facing antifungals is the development of drug resistance. Drug resistance in *C. auris* is a pertinent example of this problem. One study from 2017 reported that 93% of *C. auris* isolates analyzed displayed resistance to the azole fluconazole [19]. *C. auris* developed resistance to fluconazole through point mutations and increases in copy numbers of the gene *ERG11*, which codes for 14α-lanosterol demethylase in the ergosterol biosynthesis pathway [20]. Point mutations within *ERG11* at F126L, Y132F, and K143R are known to reduce azole susceptibility in *C. auris* [21]. Residues F126 and Y132 are within the binding site of *ERG11* and it is believed that mutations here directly reduce the binding affinity of azoles to *ERG11*. This same study found that 35% of *C. auris* isolates also displayed resistance to amphotericin B from the polyene class of antifungals [19]. Mutations within *ERG3*, another gene involved in ergosterol biosynthesis, are thought to confer resistance to amphotericin B. These mutations promote the accumulation of alternative sterols in the fungal membrane that resists the action of amphotericin B [22]. While resistance to echinocandins was rare and seen in only 7% of clinical isolates of *C. auris*, it may be the beginning of a troubling trend [19]. Caspofungin and other echinocandins inhibit β-1,3-D-glucan synthase, a critical enzyme in fungal cell wall biosynthesis [23]. Mutations at S639F in *FKS1*, a gene that encodes glucan synthase, impart echinocandin resistance in *C. auris* [24]. These specific resistances are in addition to the overexpression of gene orthologs from similar *Candida* spp. coding for major facilitator superfamily transporters and ATP transporters that are believed to reduce the efficacy of all major classes of antifungals [25,26,27]. The high rates of drug resistance, coupled with delays in properly identifying *C. auris*, and the immunocompromised populations commonly infected by it, are major obstacles in effectively treating *C. auris* infections. These factors may explain the high mortality rate of 35–60% seen in patients [9,28,29].

Widespread use of antifungals has contributed to increased rates of resistance by fungal pathogens. One major example of this phenomenon can be seen among the azoles. Azoles have been widely used in agriculture since the 1960s and have been favored for their broad-spectrum activity and low cost. Environmental exposure through agricultural use is one route through which fungal pathogens can develop resistance. Researchers have successfully cultivated isolates of triazole-resistant *A. fumigatus* from shipments of tulip bulbs originating from the Netherlands, highlighting how antifungal resistance mechanisms can travel across the globe [30]. Another study from the Netherlands examined environmental and clinical isolates of *A. fumigatus* and found that both isolates shared the same resistance mechanism and displayed genetic similarity, which may provide evidence for the theory that environmental exposure is a promoter of antifungal resistance [31]. Curtailing the overuse and application of antifungals in the environment is an issue that must be addressed globally. 

### 3.2. Antifungals in the Pipeline 

Ibrexafungerp (formerly SCY-078) is a new antifungal triterpenoid glucan synthase inhibitor that has shown *in vitro* activity against *Candida* and *Aspergillus* [32,33,34]. It is currently in six clinical trials testing the efficacy of ibrexafungerp against candidiasis caused by *Candida auris* (CARES), vulvovaginal candidiasis (VVC), and invasive pulmonary aspergillosis. Ibrexafungerp is a derivative of enfumafungin, a compound highlighted in a natural product screen that is produced by an endophytic fungi from the *Hormonema* genus [35]. The success of ibrexafungerphighlights the potential of natural product screens.

Another novel antifungal in the pipeline is the broad-spectrum drug fosmanogepix (formerly APX001). Clinical trials are currently under way testing the efficacy of fosmanogepix against invasive forms of *C. auris* and *Aspergillus*. Fosmanogepix is the prodrug form that is metabolized into the active form mangoepix (formerly APX001A). Mangoepix acts through a novel mechanism by inhibiting the enzyme glycosylphosphatidylinositol-anchored wall transfer protein 1 (Gwt1), which is required for maturation and positioning of glycosylphosphatidylinositol-anchored structural mannoproteins to the fungal cell wall [36]. 

Olorofim (formerly F901318) represents a new class of antifungals, the orotomides. This novel antifungal was developed through a small molecule screen with leads modified using medicinal chemistry. Olorofim inhibits dihydroorotate dehydrogenase, a catalyst in the pyrimidine biosynthesis pathway [37]. Olorofim was shown to be very effective against many *Aspergillus* species and invasive forms of *A. fumigatus* [37,38]. While olorofim is not a broad-spectrum antifungal, its ability to treat a wide range of *Aspergillus* species is noteworthy.

### 3.3. Antifungal Activity of Previously Investigated Plant Species

Plants historically used in traditional medicine may be a source of novel compounds that can be used for the treatment of multidrug-resistant (MDR) fungal infections. There has been extensive research into the antifungal properties of plants and their natural products. We have highlighted some plants that are commonly used in traditional medicine and/or are well studied in a laboratory setting for antifungal activity (Table 1). While there are thousands of studies published on the antifungal properties of plant extracts and plant natural products, there is a need for greater adherence to Clinical & Laboratory Standards Institute (CLSI) and European Committee on Antimicrobial Susceptibility Testing (EUCAST) guidelines for antifungal testing to ensure the rigor and reproducibility of studies.

### 3.4. Plants from Traditional Medicine as Sources of Novel Small Molecules 

To protect themselves against herbivory and infection by microbes, plants have developed a sophisticated chemical defense system composed of a diverse set of secondary metabolites. Antimicrobial secondary metabolites involved in plant innate immunity are classified based on whether they are constitutively active in plant tissues (phytoanticipins) or whether they are produced in response to microbial exposure (phytoalexins) [52]. An example of a phytoanticipins can be found in oat species from the genus *Avena* that produce saponins, such as avenacin A-1, which are concentrated in their root tips and provide fungal resistance to soil-borne microbes [53]. Plants and their diverse suite of secondary metabolites are a valuable resource that can be used to identify, isolate, and develop new chemical substrates for the formulation of innovative antifungal drugs.

There are an estimated 391,000 species of plants on Earth, and more than 28,187 of them have been used as sources of medicine by humans [54,55]. Through taking an ethnobotanical approach to drug discovery, guided by traditional knowledge of plants used as medicines, the scope of the search for novel antifungal ingredients can be strategically narrowed for inclusion in antifungal discovery screening initiatives. In addition to primary ethnobotanical studies, in which researchers interview community members about the current and recent uses of plants as medicine, other sources of relevant knowledge include historic texts that cover *materia medica* of different cultures. The discovery of artemisinin, the anti-malarial drug, isolated from sweet wormwood (*Artemisia annua* L.) by Dr. Tu Youyou, was facilitated through examination of an ancient remedy recorded in the 4th century Traditional Chinese Medicine text *A Handbook of Prescriptions for Emergencies* by Ge Hong [56]. This treatment for malaria had been used in traditional medicine for thousands of years and it was only by revisiting this knowledge that the discovery of artemisinin was possible. Viewing the issue of antifungal resistance through an ethnobotanical lens may provide the same clarity needed to discover the next lead antifungal compound.

The high humidity and climate of the tropics make fungal skin infections a common occurrence. Traditional remedies from these areas have historically used native plants to treat fungal skin infections; yet in many cases, their potential as medicines has not been scientifically assessed. One study examining plants used in Brazilian traditional medicine found that three species of plants (*Schinus terebinthifolia* Raddi, *Piper regnellii* (Miq.) C.DC., and *Rumex acetosa* L.*)* were effective against multiple *Candida* spp. at ranges from 30–60 µg/mL, and three plant species (*S. terebinthifolia*, *P. regnellii,* and *Baccharis dracunculifolia* DC.) were effective against *C. neoformans* at 30 µg/mL [57]. Tropical fruits have also been used as ingredients in traditional medicine and can act as additional sources for new small molecules to combat human fungal pathogens. Malaysia is rich in understudied tropical fruits, such as the *Mangifera pajang* Kosterm. tree. Extracts made from *M. pajang* fruit were found to be preferentially active against a variety of fungal species, exhibiting MICs of 80 µg/mL against *C. albicans*, 3 µg/mL against *C. parapsilosis*, 1 µg/mL against *C. krusei*, and 20 µg/mL against *C. neoformans* [58]. The potency and broad-spectrum activity of these extracts warrants further study aimed at the isolation and identification of the most active compounds. Filling the drug discovery pipeline with novel small molecules derived from plants is one way to address the emerging challenge of drug-resistant fungal pathogens.

### 3.5. Sourcing Plant Natural Products for Drug Discovery

Plants have been used by humans throughout history as a source of medicine. Until recently, the difficulty has been in finding, sourcing, and extracting the compounds within those plants to analyze their effects. To overcome this challenge, the National Cancer Institute (NCI) started a program to develop a publicly available high-throughput screening natural product library. From this monumental work came the NCI Program for Natural Product Discovery (NPNPD) Prefractioned Library, which has more than 150,000 natural product extracts with an aim of eventually containing 1,000,000 extract fractions [59]. The NPNPD Library is a cost-free chemical library available to scientists for testing against any disease system and target. Researchers can use this in screening campaigns to identify biologically active extracts and lead compounds for drug development. The NPNPD library removes the difficult and time-consuming burden of collecting and extracting hundreds of thousands of natural products. This library integrates the richness in biodiversity and chemical diversity found in nature and incorporates these elements into the drug discovery process.

While the NPNPD library is amenable to researchers conducting high-throughput screening, the option to travel into the field and collect bulk specimens of new, intriguing plant species will always be open to researchers. Ethical bioprospecting, via collecting field specimens, is a valuable and essential process needed to create the base from which natural product libraries, such as the NCI’s, are built on. To that end, researchers and field specialists should adhere to the articles outlined by the Convention on Biodiversity and the Nagoya Protocol, which promote the development of equitable access and benefit sharing plans for the utilization of genetic resources [60]. There are many papers and guides outlining the issues surrounding the ethical sourcing of natural products and various articles describing how to process plants; some examples are the articles by Beutler [61] and Kingston [62].

## 4. Conclusions

Traditional medicine can serve as a guide during the antifungal drug discovery process. By utilizing the knowledge of plants used as anti-infectives in systems of traditional medicine from across the globe, we can uncover species that have been overlooked by modern medicine. The plants within these time-tested traditional remedies may contain bioactive compounds effective against drug-resistant fungal pathogens.

## Figures and Tables

**Table 1:** Antifungal activity of select plant species.

Botanical Source	Common Name	Tested Sample ^1^	MIC (µg/mL)	Fungi	Ref.
*Cinnamomum cassia* (L.) J.Presl, Lauraceae	Chinese Cinnamon	EO	0.008 *	*Candida albicans*	[39]
		EO	0.031 *	*Candida glabrata*	
*Dorstenia mannii* Hook.f., Moraceae	Manpower (Bakossi)	Dorsmanin E	8	*Candida albicans*	[40]
		Dorsmanin F	16		
*Euphorbia tirucalli* L., Euphorbiaceae	Indian Tree Spurge	Aq	12.8–205.5	*Cryptococcus neoformans*	[41]
*Melaleuca alternifolia* (Maiden & Betche) Cheel, Myrtaceae	Tea Tree	EO	0.125 *	*Candida albicans*	[42]
		Terpinen-4-ol	0.06 *		
*Nigella sativa* L., Ranunculaceae	Black Caraway	EO	4	*Trichophyton mentagrophytes*	[43]
		EO	4	*Microsporum canis*	
		EO	4	*Microsporum gypseum*	
*Origanum vulgare* L., Lamiaceae	Oregano	EO	18–180	*Candida albicans*	[44]
*Panax ginseng* C.A.Mey, Araliaceae	Asian Ginseng	Ginsenosides	100	*Candida albicans*	[45]
*Ricinus communis* L., Euphorbiaceae	Castor Bean	Aq	12.5	*Candida albicans*	[46]
		Et	25		
		Me	12.5		
*Rosmarinus officinalis* L., Lamiaceae	Rosemary	Dcm	7–15	*Candida* sp.	[47]
		Me	1–7		
*Salvia ringens* Sm., Lamiaceae	Mount Olympus Sage	EO	3	*Aspergillus fumigatus*	[48]
*Syzygium aromaticum* (L.) Merr. & L.M.Perry, Myrtaceae	Clove	EO	0.64 *	*Candida albicans* *Candida albicans*	[49]
		Eugenol	0.32–0.64 *		
*Thymus vulgaris* L., Lamiaceae	Common Thyme	Thymol	39–78	*Candida* sp.	[47,50]
*Waltheria indica* L., Malvaceae	Sleepy Morning	Waltherione E	4–32	*Candida* sp.	[51]

^1^ Aq—Aqueous extract; Dcm—Dichloromethane extract; Et—Ethanol extract; Me—Methanol extract; EO—Essential oil; * Measured in percent v/v.
